# Checklist of Fishes from Madagascar Reef, Campeche Bank, México

**DOI:** 10.3897/BDJ.2.e1100

**Published:** 2014-05-08

**Authors:** Salvador Zarco Perello, Rigoberto Moreno Mendoza, Nuno Simões

**Affiliations:** † CSIRO Ecosystem Sciences, Townsville, Australia; ‡ Unidad Multidisciplinaria de Docencia e Investigación Sisal, Facultad de Ciencias, Universidad Nacional Autónoma de México, Sisal, Mexico; § Departamento de Biología Marina, Universidad Autónoma de Yucatán, Merida, Mexico

**Keywords:** Coral, reef, fishes, species richness, Mexican Atlantic, Gulf of Mexico, Campeche Bank, Yucatan, Mexico

## Abstract

This study presents the first list of fish species from Madagascar Reef, Campeche Bank, Gulf of México. Field surveys and literature review identified 54 species belonging to 8 orders, 30 families and 43 genera, comprising both conspicuous and cryptic fishes. Species richness was lower at this reef site compared to reefs in the Mexican Caribbean, Veracruz or Tuxpan, but was similar to other reefs in the same region. Species composition was a mixture of species present in all the reef systems of the Mexican Atlantic. *Hypoplectrus ecosur* was recorded here for the first time in the Gulf of Mexico, *Mycteroperca microlepis*, *Equetus lanceolatus* and *Chaetodipterus faber* were new records for the reefs of the Campeche Bank, *Elacatinus xanthiprora* was recorded for the second time in Mexico and expanded its known distribution westwards from Alacranes Reef and *Sanopus reticulatus*, endemic of the Yucatan state, was recorded here for the first time on a reef.

## Introduction

Coral reefs are important centres of fish biodiversity. About 4000 fish species are associated with coral reefs around the world (Allen 2008), representing about 25% of all the species of marine fishes known today (Nelson 2006). Fishes are one of the most diverse groups of organisms in this ecosystem (Sale 2002) and occupy all consumer trophic levels and reef habitats (Holmlund and Hammer 1999). The absence or presence of certain guilds regulate the abundance of other reef organisms, such as corals or macroalgae, and can cause drastic changes in ecosystem states (Bellwood et al. 2006).

The Mexican Atlantic is characterized by several reef ecosystems with diverse fish communities, containing about 40% of all the reef species in the Western Atlantic (Floeter et al. 2008). Mexican Caribbean reefs host approximately 393 species (Schmitter-Soto et al. 2000), while the Gulf of Mexico (GoMx), which can be divided into the Tuxpan Reef System (TRS), Veracruz Reef System (VRS) and Campeche Bank (CB), has 376 species recorded (Withers and Tunnell Jr 2007). Although the species richness of these reef systems is alike, their similarity in species composition differs as a function of their environmental conditions and their connectivity (Chávez-Hidalgo et al. 2008). The Mexican Caribbean reefs are located in a tropical environment, whilst the TRS and VRS regions are more temperate. The CB reefs lie in between, receiving waters from the Caribbean through the Yucatan Channel that then travel to the inner areas of the GoMx, reaching the VRS and TRS. Thus, the reefs in the CB could act as stepping stones between the Mexican Caribbean reefs and the TRS/VRS (Jordán-Dahlgren 2002, Villegas-Sánchez et al. 2014).

The ichthyofauna of the TRS, VRS and Mexican Caribbean has been studied extensively, while most of the CB reefs lack information. Detailed lists of species for the TRS, VRS and Mexican Caribbean have been generated and updated (Schmitter-Soto et al. 2000, Del Moral Flores et al. 2013, González-Gándara et al. 2013). In contrast, icthyological studies in the CB are scarce and limited to just a few reefs: Cayo Arcas (Garduño and Chávez 2000), Cayo Arenas (Chávez 1966, Garduño and Chávez 2000), Triangulos Oeste (Chávez 1966, Garduño and Chávez 2000) and Alacranes reef (González-Gandara and Arias-González 2001). At least nine other recognized reefs within this system do not have information about their fish communities (Tunnell Jr 2007), and a further indefinite number of reefs remain to be described even at the most basic level (Zarco-Perelló et al. 2013).

Given their proximity to the Caribbean and their closeness to the shore (Chávez 1994, Zarco-Perelló et al. 2013), these reefs are potentially important centres of biodiversity and sources of fishery products for human communities living on the coast of the Yucatan state. Thus far, only three reefs known as Sisal Reefs have being researched, and only regarding some aspects of their benthic communities (Duarte et al. 2014, González-Muñoz et al. 2013, Ortigosa et al. 2013, Santana-Moreno et al. 2013, Zarco-Perelló et al. 2013). In this study, we improve the information known about one of these reefs by providing a list of cryptic, benthic and pelagic fish species associated with Madagascar Reef. The richness and composition found is then compared with other reefs systems of the Mexican Atlantic.

## Materials and methods

### Study Site

**Figure 1.**
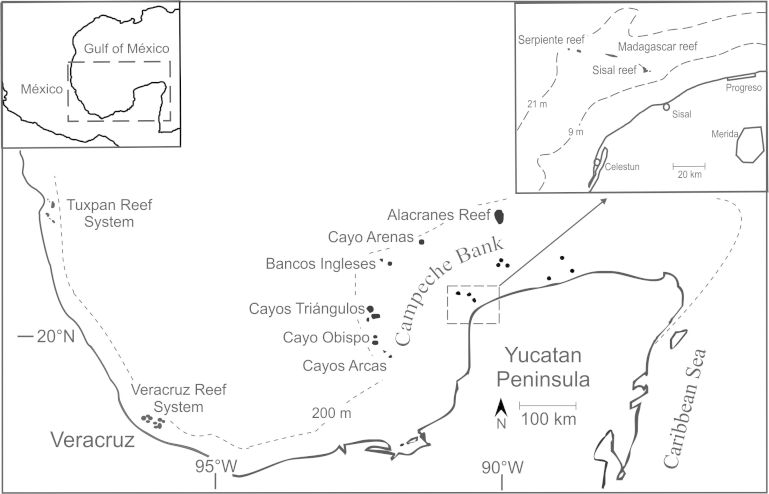
Madagascar Reef, part of the Sisal Reefs (upper-right magnifying glass), and other reefs and reef systems of the Mexican Atlantic (Modified from Jordán-Dahlgren (2002)).

Madagascar Reef is part of a cluster of three reefs named Sisal Reefs. These reefs are located in the Campeche Bank, a large carbonate platform of gentle slope (Fig. 1) that presents a low topographic complexity that is only increased sporadically by the presence of scattered reefs. Madagascar Reef is located 40 km from the fishing port of Sisal, whose inhabitants exploit its marine resources; however, fishermen from nearby ports, such as Celestun and Progreso, also visit the reef. Its morphology is peculiarly elongated, extending 2.5 km East-West and 130 m North-South in its widest point. The reef rises from sandy plains at 14m of depth to the reef crest at 4 m depth, where the illumination is high and the water current stronger (Zarco-Perelló et al. 2013).

### Data collection

**Figure 2.**
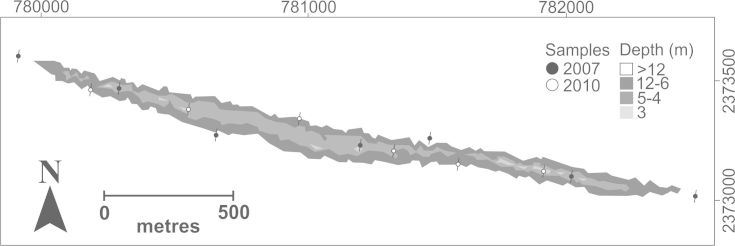
Sampling locations on Madagascar Reef. Coordinates in UTM 15N, WGS84.

Fish species occurrence was registered during two different surveys. During the first campaign (2007), seven transects of 50 m were deployed and all fishes on sight were photographed along the way for later identification (Fig. 2). The second campaign (2010) focused on cryptic species, which historically have received less attention (MacNeil et al. 2008). In this campaign, six transects of 50 m were deployed and species were counted visually and/or collected using clove oil (eugenol) diluted to 10% with 70% ethanol and sea water (Cunha and Rosa 2006). Collected fishes were preserved in 70% ethanol for later identification in the laboratory. The surveys included areas from the shallow reef crest (5 m) to the deep sandy plains around the reefs (25 m). The taxonomic identification of the species was based on Robins and Ray (1999), Böhlke and Chaplin (1993), McEachran and Fechhelm (1998), McEachran and Fechhelm (2006), Humann and DeLoach (2008). The nomenclature was revised using the Catalog of Fishes (Eschmeyer and Fricke 2013). The order of the species in the list was arranged following Nelson (2006) for the suprageneric categories, while the genera and species were ordered alphabetically. Information about the general geographic distribution of the species was based on Felder and Camp (2009), Carpenter (2002a), Carpenter (2002b) and Colin (2010) for the species of the genus *Elacatinus*. All the species collected and photographed were registered in the Ichthyology Collection (YUC-PEC-239-01-11) of the Unidad Multidiciplinaria de Docencia e Investigacion Sisal (UMDI-Sisal), a research station of the National Autonomous University of Mexico (UNAM). The list of species found in our field work was complemented with species documented in the M.Sc. thesis work of Martínez de la Portilla (2008) who surveyed Madagascar Reef in 2005.

### Species richness estimation

To evaluate if more sampling effort is needed to register all the fish species present on Madagascar Reef we calculated a species accumulation curve using the software EstimateS v.9. which utilizes a novel method developed by Colwell et al. (2004) and Colwell et al. (2012) that links rarefaction and extrapolation for presence/absence data samples.

## Checklists

### Checklist of fishes from Madagascar Reef, Campeche Bank, Mexico.

#### Urobatis jamaicensis

(Cuvier, 1816)

##### Materials

- **Type status:** Other material. **Occurrence:** catalogNumber: CIRR-302; recordedBy: Salvador Zarco Perello; individualCount: 1; **Location:** continent: America; country: Mexico; stateProvince: Yucatan; locality: Madagascar Reef; verbatimDepth: 12 m; verbatimLatitude: 781272.611854; verbatimLongitude: 2373393.69326; verbatimCoordinateSystem: UTM 15N; verbatimSRS: WGS84; decimalLatitude: 21.441017; decimalLongitude: -90.286299; **Event:** samplingProtocol: Photosampling; eventDate: 28/9/2007; **Record Level:** collectionID: YUC-PEC_239-01-64; institutionCode: UMDI-SISAL; collectionCode: CIRR

##### Distribution

Western Atlantic. North Carolina to North Brazil. Including Bermuda, Bahamas and throughout the Caribbean Islands.

#### Gymnothorax funebris

Ranzani, 1839

##### Materials

- **Type status:** Other material. **Occurrence:** catalogNumber: CIRR-292; recordedBy: Salvador Zarco Perello; individualCount: 3; **Location:** continent: America; country: Mexico; stateProvince: Yucatan; locality: Madagascar Reef; verbatimDepth: 5 m; verbatimLatitude: 780535.103072; verbatimLongitude: 2373588.16789; verbatimCoordinateSystem: UTM 15N; verbatimSRS: WGS84; decimalLatitude: 21.442888; decimalLongitude: -90.293376; **Event:** samplingProtocol: Photosampling; eventDate: 20/9/2007; **Record Level:** collectionID: YUC-PEC_239-01-64; institutionCode: UMDI-SISAL; collectionCode: CIRR

##### Distribution

Western Atlantic: Florida to Brazil. Including Bermuda, Bahamas and throughout the Caribbean Islands. Eastern Atlantic: Cape Verde, Ascension and St. Helena.

#### Synodus

Scopoli, 1777

##### Materials

- **Type status:** Other material. **Occurrence:** recordedBy: Gabriela Martinez Portilla; individualCount: 1; **Location:** continent: America; country: Mexico; stateProvince: Yucatan; locality: Madagascar Reef; verbatimLatitude: 781272.611854; verbatimLongitude: 2373443.69326; verbatimCoordinateSystem: UTM 15N; verbatimSRS: WGS84; decimalLatitude: 21.441469; decimalLongitude: -90.286290; **Event:** samplingProtocol: Visual census; eventDate: 28/7/2005

##### Distribution

Worldwide.

##### Notes

Occurrence reported by Martínez de la Portilla (2008).

#### Sanopus reticulatus

Collette, 1983

##### Materials

- **Type status:** Other material. **Occurrence:** recordedBy: Rigoberto Moreno Mendoza; individualCount: 1; **Location:** continent: America; country: Mexico; stateProvince: Yucatan; locality: Madagascar Reef; verbatimLatitude: 781272.611854; verbatimLongitude: 2373443.69326; verbatimCoordinateSystem: UTM 15N; verbatimSRS: WGS84; decimalLatitude: 21.441469; decimalLongitude: -90.286290; **Event:** samplingProtocol: Collected with clove oil; eventDate: 24/2/2010; **Record Level:** collectionID: YUC-PEC_239-01-64; institutionCode: UMDI-SISAL; collectionCode: CIRR

##### Ecological interactions

###### Conservation status

Vulnerable (IUCN).

##### Distribution

Western Atlantic. Endemic of Yucatan, Gulf of Mexico.

##### Notes

First record on a coral reef. Distribution expanded westwards from Puerto Progreso (Collette 1983).

#### Holocentrus adscensionis

(Osbeck, 1765)

##### Materials

- **Type status:** Other material. **Occurrence:** catalogNumber: CIRR-296; recordedBy: Salvador Zarco Perello; individualCount: 1; **Location:** continent: America; country: Mexico; stateProvince: Yucatan; locality: Madagascar Reef; verbatimDepth: 5m; verbatimLatitude: 782271.440297; verbatimLongitude: 2373268.56034; verbatimCoordinateSystem: UTM 15N; verbatimSRS: WGS84; decimalLatitude: 21.439732; decimalLongitude: -90.276691; **Event:** samplingProtocol: Photosampling; eventDate: 8/10/2007; **Record Level:** collectionID: YUC-PEC_239-01-64; institutionCode: UMDI-SISAL; collectionCode: CIRR

##### Distribution

Western Atlantic: Virginia to Brazil. Northwestern, northeastern, and southernGulf of Mexico, Bermuda, Bahamas and throughout the Caribbean Islands. Eastern Atlantic: Sao Tome Island and Gabon to Angola.

#### Scorpaena plumieri

Bloch, 1789

##### Materials

- **Type status:** Other material. **Occurrence:** recordedBy: Rigoberto Moreno Mendoza; individualCount: 1; **Location:** continent: America; country: Mexico; stateProvince: Yucatan; locality: Madagascar Reef; verbatimLatitude: 781272.611854; verbatimLongitude: 2373443.69326; verbatimCoordinateSystem: UTM 15N; verbatimSRS: WGS84; decimalLatitude: 21.441469; decimalLongitude: -90.286290; **Event:** samplingProtocol: Collected with clove oil; eventDate: 24/2/2010; **Record Level:** collectionID: YUC-PEC_239-01-64; institutionCode: UMDI-SISAL; collectionCode: CIRR

##### Distribution

Western Atlantic. Massachusetts to Brazil. Including Bermuda, Bahamas and throughout the Caribbean Islands.

#### Epinephelus adscensionis

(Osbeck, 1765)

##### Materials

- **Type status:** Other material. **Occurrence:** catalogNumber: CIRR-290; recordedBy: Salvador Zarco Perello; individualCount: 3; **Location:** continent: America; country: Mexico; stateProvince: Yucatan; locality: Madagascar Reef; verbatimDepth: 5 m; verbatimLatitude: 781272.611854; verbatimLongitude: 2373443.69326; verbatimCoordinateSystem: UTM 15N; verbatimSRS: WGS84; decimalLatitude: 21.441469; decimalLongitude: -90.286290; **Event:** samplingProtocol: Photosampling; eventDate: 8/10/2007; **Record Level:** collectionID: YUC-PEC_239-01-64; institutionCode: UMDI-SISAL; collectionCode: CIRR

##### Distribution

Western Atlantic: Massachusetts to South Brazil. Including Bermuda, Bahamas and throughout the Caribbean Islands. Eastern Atlantic: St. Helena, Ascension Island and São Tomé.

#### Epinephelus morio

(Valenciennes, 1828)

##### Materials

- **Type status:** Other material. **Occurrence:** recordedBy: Rigoberto Moreno Mendoza; individualCount: 1; **Location:** continent: America; country: Mexico; stateProvince: Yucatan; locality: Madagascar Reef; verbatimLatitude: 781272.611854; verbatimLongitude: 2373443.69326; verbatimCoordinateSystem: UTM 15N; verbatimSRS: WGS84; decimalLatitude: 21.441469; decimalLongitude: -90.286290; **Event:** samplingProtocol: Photosampling; eventDate: 24/2/2010

##### Distribution

Western Atlantic. North Carolina to South Brazil. Including Bermuda, Bahamas and throughout the Caribbean Islands.

#### Hypoplectrus ecosur

Victor, 2012

##### Materials

- **Type status:** Other material. **Occurrence:** catalogNumber: CIRR-297; recordedBy: Salvador Zarco Perello; individualCount: 2; **Location:** continent: America; country: Mexico; stateProvince: Yucatan; locality: Madagascar Reef; verbatimDepth: 7 m; verbatimLatitude: 780143.766831; verbatimLongitude: 2373700.0708; verbatimCoordinateSystem: UTM 15N; verbatimSRS: WGS84; decimalLatitude: 21.443959; decimalLongitude: -90.297130; **Event:** samplingProtocol: Photosampling; eventDate: 24/9/2007; **Record Level:** collectionID: YUC-PEC_239-01-64; institutionCode: UMDI-SISAL; collectionCode: CIRR

##### Distribution

Western Atlantic. Mexico, Contoy Island to Campeche Bank.

##### Notes

First record in the Gulf of Mexico (Victor 2012).

#### Mycteroperca bonaci

(Poey, 1860)

##### Materials

- **Type status:** Other material. **Occurrence:** recordedBy: Gabriela Martinez Portilla; individualCount: 1; **Location:** continent: America; country: Mexico; stateProvince: Yucatan; locality: Madagascar Reef; verbatimLatitude: 781272.611854; verbatimLongitude: 2373443.69326; verbatimCoordinateSystem: UTM 15N; verbatimSRS: WGS84; decimalLatitude: 21.441469; decimalLongitude: -90.286290; **Event:** samplingProtocol: Visual census; eventDate: 13/5/2005

##### Distribution

Western Atlantic. Florida Keys and Gulf of Mexico to Brazil. Including Bermuda, Bahamas and throughout the Caribbean Islands.

##### Notes

Occurrence reported by Martínez de la Portilla (2008).

#### Mycteroperca microlepis

(Goode & Bean, 1879)

##### Materials

- **Type status:** Other material. **Occurrence:** recordedBy: Rigoberto Moreno Mendoza; individualCount: 1; **Location:** continent: America; country: Mexico; stateProvince: Yucatan; locality: Madagascar Reef; verbatimLatitude: 781272.611854; verbatimLongitude: 2373443.69326; verbatimCoordinateSystem: UTM 15N; verbatimSRS: WGS84; decimalLatitude: 21.441469; decimalLongitude: -90.286290; **Event:** samplingProtocol: Visual census; eventDate: 24/2/2010

##### Distribution

Western Atlantic. North Carolina to Yucatan Peninsula (Gulf of Mexico), including Cuba. Also reported in Brazil.

##### Notes

First record on a Campeche Bank reef.

#### Mycteroperca venenosa

(Linnaeus, 1758)

##### Materials

- **Type status:** Other material. **Occurrence:** recordedBy: Gabriela Martinez Portilla; individualCount: 1; **Location:** continent: America; country: Mexico; stateProvince: Yucatan; locality: Madagascar Reef; verbatimLatitude: 781272.611854; verbatimLongitude: 2373443.69326; verbatimCoordinateSystem: UTM 15N; verbatimSRS: WGS84; decimalLatitude: 21.441469; decimalLongitude: -90.286290; **Event:** samplingProtocol: Visual census; eventDate: 13/5/2005

##### Distribution

Western Atlantic. North Carolina, south Florida, Gulf of Mexico (rare), Honduras, Nicaragua and from Venezuela to São Paulo, Brazil. Including Bermuda, Bahamas and throughout the Caribbean Islands.

##### Notes

Occurrence reported by Martínez de la Portilla (2008).

#### Serranus subligarius

(Cope, 1870)

##### Materials

- **Type status:** Other material. **Occurrence:** recordedBy: Gabriela Martinez Portilla; individualCount: 1; **Location:** continent: America; country: Mexico; stateProvince: Yucatan; locality: Madagascar Reef; verbatimLatitude: 781272.611854; verbatimLongitude: 2373443.69326; verbatimCoordinateSystem: UTM 15N; verbatimSRS: WGS84; decimalLatitude: 21.441469; decimalLongitude: -90.286290; **Event:** samplingProtocol: Visual census; eventDate: 13/5/2005

##### Distribution

Western Atlantic. North Carolina to Gulf of Mexico.

##### Notes

Occurrence reported by Martínez de la Portilla (2008).

#### Opistognathus aurifrons

(Jordan & Thompson, 1905)

##### Materials

- **Type status:** Other material. **Occurrence:** catalogNumber: CIRR-250; recordedBy: Rigoberto Moreno Mendoza; individualCount: 1; **Location:** continent: America; country: Mexico; stateProvince: Yucatan; locality: Madagascar Reef; verbatimLatitude: 781272.611854; verbatimLongitude: 2373443.69326; verbatimCoordinateSystem: UTM 15N; verbatimSRS: WGS84; decimalLatitude: 21.441469; decimalLongitude: -90.286290; **Event:** samplingProtocol: Collected with clove oil; eventDate: 24/2/2010; **Record Level:** collectionID: YUC-PEC_239-01-64; institutionCode: UMDI-SISAL; collectionCode: CIRR

##### Distribution

Western Atlantic. Florida to Central America. Including Bahamas.

#### Astrapogon stellatus

(Cope, 1867)

##### Materials

- **Type status:** Other material. **Occurrence:** catalogNumber: CIRR-243; recordedBy: Rigoberto Moreno Mendoza; individualCount: 1; **Location:** continent: America; country: Mexico; stateProvince: Yucatan; locality: Madagascar Reef; verbatimLatitude: 781272.611854; verbatimLongitude: 2373443.69326; verbatimCoordinateSystem: UTM 15N; verbatimSRS: WGS84; decimalLatitude: 21.441469; decimalLongitude: -90.286290; **Event:** samplingProtocol: Collected with clove oil; eventDate: 24/2/2010; **Record Level:** collectionID: YUC-PEC_239-01-64; institutionCode: UMDI-SISAL; collectionCode: CIRR

##### Distribution

Western Atlantic. Florida to Venezuela. Including Bermuda and Bahamas.

#### Echeneis

Linnaeus, 1758

##### Materials

- **Type status:** Other material. **Occurrence:** recordedBy: Salvador Zarco Perello; individualCount: 1; **Location:** continent: America; country: Mexico; stateProvince: Yucatan; locality: Madagascar Reef; verbatimDepth: 5 m; verbatimLatitude: 780535.103072; verbatimLongitude: 2373588.16789; verbatimCoordinateSystem: UTM 15N; verbatimSRS: WGS84; decimalLatitude: 21.442888; decimalLongitude: -90.293376; **Event:** samplingProtocol: Photosampling; eventDate: 24/9/2007; **Record Level:** collectionID: YUC-PEC_239-01-64; institutionCode: UMDI-SISAL; collectionCode: CIRR

##### Distribution

Worldwide.

#### Carangoides ruber

(Bloch, 1793)

##### Materials

- **Type status:** Other material. **Occurrence:** catalogNumber: CIRR-289; recordedBy: Salvador Zarco Perello; individualCount: 5; **Location:** continent: America; country: Mexico; stateProvince: Yucatan; locality: Madagascar Reef; verbatimDepth: 7 m; verbatimLatitude: 780143.766831; verbatimLongitude: 2373680.0708; verbatimCoordinateSystem: UTM 15N; verbatimSRS: WGS84; decimalLatitude: 21.443778; decimalLongitude: -90.297133; **Event:** samplingProtocol: Photosampling; eventDate: 24/9/2007; **Record Level:** collectionID: YUC-PEC_239-01-64; institutionCode: UMDI-SISAL; collectionCode: CIRR

##### Distribution

Western Atlantic. New Jersey to Venezuela. Including Bermuda, Bahamas and throughout the Caribbean Islands.

#### Lutjanus apodus

(Walbaum, 1792)

##### Materials

- **Type status:** Other material. **Occurrence:** recordedBy: Gabriela Martinez Portilla; individualCount: 1; **Location:** continent: America; country: Mexico; stateProvince: Yucatan; locality: Madagascar Reef; verbatimLatitude: 781272.611854; verbatimLongitude: 2373443.69326; verbatimCoordinateSystem: UTM 15N; verbatimSRS: WGS84; decimalLatitude: 21.441469; decimalLongitude: -90.286290; **Event:** samplingProtocol: Visual census; eventDate: 13/5/2005

##### Distribution

Western Atlantic. Massachusetts to North Brazil. Including Bermuda, Bahamas and throughout the Caribbean Islands.

##### Notes

Occurrence reported by Martínez de la Portilla (2008).

#### Lutjanus griseus

(Linnaeus, 1758)

##### Materials

- **Type status:** Other material. **Occurrence:** catalogNumber: CIRR-298; recordedBy: Salvador Zarco Perello; individualCount: 10; **Location:** continent: America; country: Mexico; stateProvince: Yucatan; locality: Madagascar Reef; verbatimDepth: 15 m; verbatimLatitude: 781731.820967; verbatimLongitude: 2373387.22376; verbatimCoordinateSystem: UTM 15N; verbatimSRS: WGS84; decimalLatitude: 21.440887; decimalLongitude: -90.281873; **Event:** samplingProtocol: Photosampling; eventDate: 11/9/2007; **Record Level:** collectionID: YUC-PEC_239-01-64; institutionCode: UMDI-SISAL; collectionCode: CIRR

##### Distribution

Western Atlantic. Massachusetts to South Brazil. Including Bermuda, Bahamas and throughout the Caribbean Islands. Also reported in the eastern Atlantic off west Africa.

#### Ocyurus chrysurus

(Bloch, 1791)

##### Materials

- **Type status:** Other material. **Occurrence:** catalogNumber: CIRR-299; recordedBy: Salvador Zarco Perello; individualCount: 2; **Location:** continent: America; country: Mexico; stateProvince: Yucatan; locality: Madagascar Reef; verbatimDepth: 15 m; verbatimLatitude: 780535.103072; verbatimLongitude: 2373588.16789; verbatimCoordinateSystem: UTM 15N; verbatimSRS: WGS84; decimalLatitude: 21.442888; decimalLongitude: -90.293376; **Event:** samplingProtocol: Photosampling; eventDate: 20/9/2007; **Record Level:** collectionID: YUC-PEC_239-01-64; institutionCode: UMDI-SISAL; collectionCode: CIRR

##### Distribution

Western Atlantic. Massachusetts to South Brazil. Including Bermuda, Bahamas and throughout the Caribbean Islands. Eastern Atlantic: Cape Verde.

#### Anisotremus virginicus

(Linnaeus, 1758)

##### Materials

- **Type status:** Other material. **Occurrence:** catalogNumber: CIRR-288; recordedBy: Salvador Zarco Perello; individualCount: 50; **Location:** continent: America; country: Mexico; stateProvince: Yucatan; locality: Madagascar Reef; verbatimDepth: 15 m; verbatimLatitude: 781731.820967; verbatimLongitude: 2373387.22376; verbatimCoordinateSystem: UTM 15N; verbatimSRS: WGS84; decimalLatitude: 21.440887; decimalLongitude: -90.281873; **Event:** samplingProtocol: Photosampling; eventDate: 11/9/2007; **Record Level:** collectionID: YUC-PEC_239-01-64; institutionCode: UMDI-SISAL; collectionCode: CIRR

##### Distribution

Western Atlantic. Florida to Brazil. Including Bermuda, Bahamas and throughout the Caribbean Islands.

#### Haemulon aurolineatum

Cuvier, 1830

##### Materials

- **Type status:** Other material. **Occurrence:** catalogNumber: CIRR-293; recordedBy: Salvador Zarco Perello; individualCount: 40; **Location:** continent: America; country: Mexico; stateProvince: Yucatan; locality: Madagascar Reef; verbatimDepth: 5 m; verbatimLatitude: 782271.440297; verbatimLongitude: 2373268.56034; verbatimCoordinateSystem: UTM 15N; verbatimSRS: WGS84; decimalLatitude: 21.439732; decimalLongitude: -90.276691; **Event:** samplingProtocol: Photosampling; eventDate: 8/10/2007; **Record Level:** collectionID: YUC-PEC_239-01-64; institutionCode: UMDI-SISAL; collectionCode: CIRR

##### Distribution

Western Atlantic. Virginia to Brazil. Including Bermuda, Bahamas and throughout the Caribbean Islands.

#### Haemulon plumierii

(Lacepède, 1801)

##### Materials

- **Type status:** Other material. **Occurrence:** recordedBy: Rigoberto Moreno Mendoza; individualCount: 1; **Location:** continent: America; country: Mexico; stateProvince: Yucatan; locality: Madagascar Reef; verbatimLatitude: 781272.611854; verbatimLongitude: 2373443.69326; verbatimCoordinateSystem: UTM 15N; verbatimSRS: WGS84; decimalLatitude: 21.441469; decimalLongitude: -90.286290; **Event:** samplingProtocol: Photosampling; eventDate: 13/5/2005

##### Distribution

Western Atlantic. Virginia to Brazil. Including Bermuda, Bahamas and throughout the Caribbean Islands.

#### Calamus

(Valenciennes, 1830)

##### Materials

- **Type status:** Other material. **Occurrence:** recordedBy: Salvador Zarco Perello; individualCount: 7; **Location:** continent: America; country: Mexico; stateProvince: Yucatan; locality: Madagascar Reef; verbatimDepth: 7 m; verbatimLatitude: 780143.766831; verbatimLongitude: 2373680.0708; verbatimCoordinateSystem: UTM 15N; verbatimSRS: WGS84; decimalLatitude: 21.443778; decimalLongitude: -90.297133; **Event:** samplingProtocol: Photosampling; eventDate: 20/9/2007; **Record Level:** collectionID: YUC-PEC_239-01-64; institutionCode: UMDI-SISAL; collectionCode: CIRR

##### Distribution

Worldwide.

#### Equetus lanceolatus

(Linnaeus, 1758)

##### Materials

- **Type status:** Other material. **Occurrence:** catalogNumber: CIRR-291; recordedBy: Salvador Zarco Perello; individualCount: 2; **Location:** continent: America; country: Mexico; stateProvince: Yucatan; locality: Madagascar Reef; verbatimDepth: 17 m; verbatimLatitude: 780143.766831; verbatimLongitude: 2373740.0708; verbatimCoordinateSystem: UTM 15N; verbatimSRS: WGS84; decimalLatitude: 21.444320; decimalLongitude: -90.297123; **Event:** samplingProtocol: Photosampling; eventDate: 24/9/2007; **Record Level:** collectionID: YUC-PEC_239-01-64; institutionCode: UMDI-SISAL; collectionCode: CIRR

##### Distribution

Western Atlantic. South Carolina to Brazil. Including Bermuda, Bahamas and throughout the Caribbean Islands.

##### Notes

First record on a Campeche Bank reef.

#### Pareques umbrosus

(Jordan & Eigenmann, 1889)

##### Materials

- **Type status:** Other material. **Occurrence:** catalogNumber: CIRR-300; recordedBy: Salvador Zarco Perello; individualCount: 5; **Location:** continent: America; country: Mexico; stateProvince: Yucatan; locality: Madagascar Reef; verbatimDepth: 16 m; verbatimLatitude: 780535.103072; verbatimLongitude: 2373673.16789; verbatimCoordinateSystem: UTM 15N; verbatimSRS: WGS84; decimalLatitude: 21.443655; decimalLongitude: -90.293362; **Event:** samplingProtocol: Photosampling; eventDate: 20/9/2007; **Record Level:** collectionID: YUC-PEC_239-01-64; institutionCode: UMDI-SISAL; collectionCode: CIRR

##### Distribution

Western Atlantic. Virginia to Brazil. Including Bermuda, Bahamas and throughout the Caribbean Islands.

#### Chaetodon ocellatus

Bloch, 1787

##### Materials

- **Type status:** Other material. **Occurrence:** recordedBy: Rigoberto Moreno Mendoza; individualCount: 1; **Location:** continent: America; country: Mexico; stateProvince: Yucatan; locality: Madagascar Reef; verbatimLatitude: 781272.611854; verbatimLongitude: 2373443.69326; verbatimCoordinateSystem: UTM 15N; verbatimSRS: WGS84; decimalLatitude: 21.441469; decimalLongitude: -90.286290; **Event:** samplingProtocol: Photosampling; eventDate: 13/5/2005

##### Distribution

Western Atlantic. Maine to Brazil. Including Bermuda, Bahamas and throughout the Caribbean Islands.

#### Holacanthus bermudensis

Goode, 1876

##### Materials

- **Type status:** Other material. **Occurrence:** catalogNumber: CIRR-295; recordedBy: Salvador Zarco Perello; individualCount: 10; **Location:** continent: America; country: Mexico; stateProvince: Yucatan; locality: Madagascar Reef; verbatimDepth: 5 m; verbatimLatitude: 782271.440297; verbatimLongitude: 2373268.56034; verbatimCoordinateSystem: UTM 15N; verbatimSRS: WGS84; decimalLatitude: 21.439732; decimalLongitude: -90.276691; **Event:** samplingProtocol: Photosampling; eventDate: 28/9/2007; **Record Level:** collectionID: YUC-PEC_239-01-64; institutionCode: UMDI-SISAL; collectionCode: CIRR

##### Distribution

Western Atlantic. Florida, Gulf of Mexico, Bahamas and Bermuda.

#### Holacanthus ciliaris

(Linnaeus, 1758)

##### Materials

- **Type status:** Other material. **Occurrence:** recordedBy: Gabriela Martinez Portilla; individualCount: 1; **Location:** continent: America; country: Mexico; stateProvince: Yucatan; locality: Madagascar Reef; verbatimLatitude: 781272.611854; verbatimLongitude: 2373443.69326; verbatimCoordinateSystem: UTM 15N; verbatimSRS: WGS84; decimalLatitude: 21.441469; decimalLongitude: -90.286290; **Event:** samplingProtocol: Visual census; eventDate: 13/5/2005

##### Distribution

Western Atlantic. Florida to Brazil. Including Bermuda, Bahamas and throughout the Caribbean Islands.

##### Notes

Occurrence reported by Martínez de la Portilla (2008).

#### Pomacanthus arcuatus

(Linnaeus, 1758)

##### Materials

- **Type status:** Other material. **Occurrence:** catalogNumber: CIRR-304; recordedBy: Salvador Zarco Perello; individualCount: 3; **Location:** continent: America; country: Mexico; stateProvince: Yucatan; locality: Madagascar Reef; verbatimDepth: 4 m; verbatimLatitude: 781272.611854; verbatimLongitude: 2373443.69326; verbatimCoordinateSystem: UTM 15N; verbatimSRS: WGS84; decimalLatitude: 21.441469; decimalLongitude: -90.286290; **Event:** samplingProtocol: Photosampling; eventDate: 28/9/2007; **Record Level:** collectionID: YUC-PEC_239-01-64; institutionCode: UMDI-SISAL; collectionCode: CIRR

##### Distribution

Western Atlantic. New York to Brazil. Including Bahamas and throughout the Caribbean Islands.

#### Abudefduf saxatilis

(Linnaeus 1758)

##### Materials

- **Type status:** Other material. **Occurrence:** catalogNumber: CIRR-284; recordedBy: Salvador Zarco Perello; individualCount: 2; **Location:** continent: America; country: Mexico; stateProvince: Yucatan; locality: Madagascar Reef; verbatimDepth: 5 m; verbatimLatitude: 782271.440297; verbatimLongitude: 2373332.34712; verbatimCoordinateSystem: UTM 15N; verbatimSRS: WGS84; decimalLatitude: 21.440307; decimalLongitude: -90.276681; **Event:** samplingProtocol: Photosampling; eventDate: 8/10/2007; **Record Level:** collectionID: YUC-PEC_239-01-64; institutionCode: UMDI-SISAL; collectionCode: CIRR

##### Distribution

Western Atlantic. North Carolina to Brazil. Including Bermuda, Bahamas and throughout the Caribbean Islands.

#### Stegastes variabilis

(Castelnau, 1855)

##### Materials

- **Type status:** Other material. **Occurrence:** catalogNumber: CIRR-311; recordedBy: Salvador Zarco Perello; individualCount: 3; **Location:** continent: America; country: Mexico; stateProvince: Yucatan; locality: Madagascar Reef; verbatimDepth: 5 m; verbatimLatitude: 780535.103072; verbatimLongitude: 2373588.16789; verbatimCoordinateSystem: UTM 15N; verbatimSRS: WGS84; decimalLatitude: 21.442888; decimalLongitude: -90.293376; **Event:** samplingProtocol: Photosampling; eventDate: 20/9/2007; **Record Level:** collectionID: YUC-PEC_239-01-64; institutionCode: UMDI-SISAL; collectionCode: CIRR

##### Distribution

Western Atlantic. North Carolina to Brazil. Including Bermuda, Bahamas and throughout the Caribbean Islands.

#### Kyphosus sectatrix

(Linnaeus 1758)

##### Materials

- **Type status:** Other material. **Occurrence:** recordedBy: Gabriela Martinez Portilla; individualCount: 1; **Location:** continent: America; country: Mexico; stateProvince: Yucatan; locality: Madagascar Reef; verbatimLatitude: 781272.611854; verbatimLongitude: 2373443.69326; verbatimCoordinateSystem: UTM 15N; verbatimSRS: WGS84; decimalLatitude: 21.441469; decimalLongitude: -90.286290; **Event:** samplingProtocol: Visual census; eventDate: 28/7/2005

##### Distribution

Western Atlantic: Maine to Brazil. Including Bermuda, Bahamas and throughout the Caribbean Islands. Eastern Atlantic: From Spain to Angola.

##### Notes

Occurrence reported by Martínez de la Portilla (2008).

#### Halichoeres

Rüppell, 1835

##### Materials

- **Type status:** Other material. **Occurrence:** recordedBy: Gabriela Martinez Portilla; individualCount: 1; **Location:** continent: America; country: Mexico; stateProvince: Yucatan; locality: Madagascar Reef; verbatimLatitude: 781272.611854; verbatimLongitude: 2373443.69326; verbatimCoordinateSystem: UTM 15N; verbatimSRS: WGS84; decimalLatitude: 21.441469; decimalLongitude: -90.286290; **Event:** samplingProtocol: Visual census; eventDate: 13/5/2005

##### Distribution

Worldwide.

##### Notes

Occurrence reported by Martínez de la Portilla (2008).

#### Lachnolaimus maximus

(Walbaum, 1792)

##### Materials

- **Type status:** Other material. **Occurrence:** catalogNumber: CIRR-303; recordedBy: Salvador Zarco Perello; individualCount: 2; **Location:** continent: America; country: Mexico; stateProvince: Yucatan; locality: Madagascar Reef; verbatimDepth: 5 m; verbatimLatitude: 780535.103072; verbatimLongitude: 2373608.16789; verbatimCoordinateSystem: UTM 15N; verbatimSRS: WGS84; decimalLatitude: 21.443068; decimalLongitude: -90.293373; **Event:** samplingProtocol: Photosampling; eventDate: 20/9/2007; **Record Level:** collectionID: YUC-PEC_239-01-64; institutionCode: UMDI-SISAL; collectionCode: CIRR

##### Distribution

Western Atlantic. North Carolina to Brazil. Including Bermuda, Bahamas and throughout the Caribbean Islands.

#### Thalassoma bifasciatum

(Bloch, 1791)

##### Materials

- **Type status:** Other material. **Occurrence:** catalogNumber: CIRR-313; recordedBy: Salvador Zarco Perello; individualCount: 2; **Location:** continent: America; country: Mexico; stateProvince: Yucatan; locality: Madagascar Reef; verbatimDepth: 10 m; verbatimLatitude: 781272.611854; verbatimLongitude: 2373443.69326; verbatimCoordinateSystem: UTM 15N; verbatimSRS: WGS84; decimalLatitude: 21.441469; decimalLongitude: -90.286290; **Event:** samplingProtocol: Photosampling; eventDate: 20/9/2007; **Record Level:** collectionID: YUC-PEC_239-01-64; institutionCode: UMDI-SISAL; collectionCode: CIRR

##### Distribution

Western Atlantic. Florida to Venezuela. Including Bermuda, Bahamas and throughout the Caribbean Islands.

#### Scarus coeruleus

(Edwards, 1771)

##### Materials

- **Type status:** Other material. **Occurrence:** catalogNumber: CIRR-306; recordedBy: Salvador Zarco Perello; individualCount: 5; **Location:** continent: America; country: Mexico; stateProvince: Yucatan; locality: Madagascar Reef; verbatimDepth: 15 m; verbatimLatitude: 782461.025319; verbatimLongitude: 2373300.35196; verbatimCoordinateSystem: UTM 15N; verbatimSRS: WGS84; decimalLatitude: 21.439989; decimalLongitude: -90.274859; **Event:** samplingProtocol: Photosampling; eventDate: 13/9/2007; **Record Level:** collectionID: YUC-PEC_239-01-64; institutionCode: UMDI-SISAL; collectionCode: CIRR

##### Distribution

Western Atlantic. Maryland to Brazil, excluding western Gulf of Mexico. Including Bermuda, Bahamas, and throughout the Caribbean Islands.

#### Sparisoma aurofrenatum

(Valenciennes, 1840)

##### Materials

- **Type status:** Other material. **Occurrence:** catalogNumber: CIRR-309; recordedBy: Salvador Zarco Perello; individualCount: 5; **Location:** continent: America; country: Mexico; stateProvince: Yucatan; locality: Madagascar Reef; verbatimDepth: 5 m; verbatimLatitude: 782271.440297; verbatimLongitude: 2373268.56034; verbatimCoordinateSystem: UTM 15N; verbatimSRS: WGS84; decimalLatitude: 21.439732; decimalLongitude: -90.276691; **Event:** samplingProtocol: Photosampling; eventDate: 8/10/2007; **Record Level:** collectionID: YUC-PEC_239-01-64; institutionCode: UMDI-SISAL; collectionCode: CIRR

##### Distribution

Western Atlantic. South Florida to Brazil, excluding western Gulf of Mexico. Including Bermuda, Bahamas, and throughout the Caribbean Islands.

#### Sparisoma rubripinne

(Valenciennes, 1840)

##### Materials

- **Type status:** Other material. **Occurrence:** catalogNumber: CIRR-308; recordedBy: Salvador Zarco Perello; individualCount: 6; **Location:** continent: America; country: Mexico; stateProvince: Yucatan; locality: Madagascar Reef; verbatimDepth: 5 m; verbatimLatitude: 782271.440297; verbatimLongitude: 2373268.56034; verbatimCoordinateSystem: UTM 15N; verbatimSRS: WGS84; decimalLatitude: 21.439732; decimalLongitude: -90.276691; **Event:** samplingProtocol: Photosampling; eventDate: 8/10/2007; **Record Level:** collectionID: YUC-PEC_239-01-64; institutionCode: UMDI-SISAL; collectionCode: CIRR

##### Distribution

Western Atlantic. Massachusetts to Brazil. Including Bermuda, Bahamas, and throughout the Caribbean Islands. Also reported in the eastern Atlantic off west Africa.

#### Sparisoma viride

(Bonnaterre, 1788)

##### Materials

- **Type status:** Other material. **Occurrence:** catalogNumber: CIRR-310; recordedBy: Salvador Zarco Perello; individualCount: 2; **Location:** continent: America; country: Mexico; stateProvince: Yucatan; locality: Madagascar Reef; verbatimDepth: 7 m; verbatimLatitude: 780143.766831; verbatimLongitude: 2373680.0708; verbatimCoordinateSystem: UTM 15N; verbatimSRS: WGS84; decimalLatitude: 21.443778; decimalLongitude: -90.297133; **Event:** samplingProtocol: Photosampling; eventDate: 24/9/2007; **Record Level:** collectionID: YUC-PEC_239-01-64; institutionCode: UMDI-SISAL; collectionCode: CIRR

##### Distribution

Western Atlantic. South Florida to Brazil. Including Bermuda, Bahamas, and throughout the Caribbean Islands.

#### Malacoctenus triangulatus

Springer, 1959

##### Materials

- **Type status:** Other material. **Occurrence:** recordedBy: Rigoberto Moreno Mendoza; individualCount: 1; **Location:** continent: America; country: Mexico; stateProvince: Yucatan; locality: Madagascar Reef; verbatimLatitude: 781272.611854; verbatimLongitude: 2373443.69326; verbatimCoordinateSystem: UTM 15N; verbatimSRS: WGS84; decimalLatitude: 21.441469; decimalLongitude: -90.286290; **Event:** samplingProtocol: Collected with clove oil; eventDate: 24/2/2010; **Record Level:** collectionID: YUC-PEC_239-01-64; institutionCode: UMDI-SISAL; collectionCode: CIRR

##### Distribution

Western Atlantic. South Florida to Brazil. Including the Caribbean Islands.

#### Parablennius marmoreus

(Poey, 1876)

##### Materials

- **Type status:** Other material. **Occurrence:** catalogNumber: CIRR-253; recordedBy: Rigoberto Moreno Mendoza; individualCount: 1; **Location:** continent: America; country: Mexico; stateProvince: Yucatan; locality: Madagascar Reef; verbatimLatitude: 781272.611854; verbatimLongitude: 2373443.69326; verbatimCoordinateSystem: UTM 15N; verbatimSRS: WGS84; decimalLatitude: 21.441469; decimalLongitude: -90.286290; **Event:** samplingProtocol: Collected with clove oil; eventDate: 24/2/2010; **Record Level:** collectionID: YUC-PEC_239-01-64; institutionCode: UMDI-SISAL; collectionCode: CIRR

##### Distribution

Western Atlantic. New York to South America. Including Bermuda, Bahamas, and throughout the Caribbean Island.

#### Coryphopterus dicrus

Böhlke & Robins, 1960

##### Materials

- **Type status:** Other material. **Occurrence:** recordedBy: Rigoberto Moreno Mendoza; individualCount: 2; **Location:** continent: America; country: Mexico; stateProvince: Yucatan; locality: Madagascar Reef; verbatimLatitude: 781272.611854; verbatimLongitude: 2373443.69326; verbatimCoordinateSystem: UTM 15N; verbatimSRS: WGS84; decimalLatitude: 21.441469; decimalLongitude: -90.286290; **Event:** samplingProtocol: Collected with clove oil; eventDate: 24/2/2010; **Record Level:** collectionID: YUC-PEC_239-01-64; institutionCode: UMDI-SISAL; collectionCode: CIRR

##### Distribution

Western Atlantic. South Florida to South America. Including Bahamas, and throughout the Caribbean Islands.

#### Coryphopterus glaucofraenum

Gill, 1863

##### Materials

- **Type status:** Other material. **Occurrence:** recordedBy: Rigoberto Moreno Mendoza; individualCount: 1; **Location:** continent: America; country: Mexico; stateProvince: Yucatan; locality: Madagascar Reef; verbatimLatitude: 781272.611854; verbatimLongitude: 2373443.69326; verbatimCoordinateSystem: UTM 15N; verbatimSRS: WGS84; decimalLatitude: 21.441469; decimalLongitude: -90.286290; **Event:** samplingProtocol: Collected with clove oil; eventDate: 24/2/2010; **Record Level:** collectionID: YUC-PEC_239-01-64; institutionCode: UMDI-SISAL; collectionCode: CIRR

##### Distribution

Western Atlantic. North Carolina to Brazil. Including Bermuda, Bahamas, and throughout the Caribbean Islands.

#### Elacatinus oceanops

Jordan, 1904

##### Materials

- **Type status:** Other material. **Occurrence:** catalogNumber: CIRR-312; recordedBy: Salvador Zarco Perello; individualCount: 3; **Location:** continent: America; country: Mexico; stateProvince: Yucatan; locality: Madagascar Reef; verbatimDepth: 5 m; verbatimLatitude: 780535.103072; verbatimLongitude: 2373588.16789; verbatimCoordinateSystem: UTM 15N; verbatimSRS: WGS84; decimalLatitude: 21.442888; decimalLongitude: -90.293376; **Event:** samplingProtocol: Photosampling; eventDate: 20/9/2007; **Record Level:** collectionID: YUC-PEC_239-01-64; institutionCode: UMDI-SISAL; collectionCode: CIRR

##### Distribution

Western Atlantic. Gulf of Mexico, east coast of Florida, north to North Carolina.

#### Elacatinus xanthiprora

(Böhlke & Robins, 1968)

##### Materials

- **Type status:** Other material. **Occurrence:** catalogNumber: CIRR-283; recordedBy: Rigoberto Moreno Mendoza; individualCount: 1; **Location:** continent: America; country: Mexico; stateProvince: Yucatan; locality: Madagascar Reef; verbatimLatitude: 781272.611854; verbatimLongitude: 2373443.69326; verbatimCoordinateSystem: UTM 15N; verbatimSRS: WGS84; decimalLatitude: 21.441469; decimalLongitude: -90.286290; **Event:** samplingProtocol: Collected with clove oil; eventDate: 24/2/2010; **Record Level:** collectionID: YUC-PEC_239-01-64; institutionCode: UMDI-SISAL; collectionCode: CIRR

##### Distribution

Western Atlantic. North Carolina, south Florida, west coast of Florida, Campeche Bank, shelf edge off Nicaragua.

##### Notes

Distribution expanded westwards from Alacranes Reef (González-Gandara and Arias-González 2001; Colin 2010).

#### Tigrigobius macrodon

(Beebe & Tee-Van, 1928)

##### Materials

- **Type status:** Other material. **Occurrence:** recordedBy: Rigoberto Moreno Mendoza; individualCount: 1; **Location:** continent: America; country: Mexico; stateProvince: Yucatan; locality: Madagascar Reef; verbatimLatitude: 781272.611854; verbatimLongitude: 2373443.69326; verbatimCoordinateSystem: UTM 15N; verbatimSRS: WGS84; decimalLatitude: 21.441469; decimalLongitude: -90.286290; **Event:** samplingProtocol: Collected with clove oil; eventDate: 24/2/2010; **Record Level:** collectionID: YUC-PEC_239-01-64; institutionCode: UMDI-SISAL; collectionCode: CIRR

##### Distribution

Western Atlantic. South Florida, Gulf of Mexico, Bermuda, Cuba to Haiti.

#### Ptereleotris calliura

(Jordan & Gilbert, 1882)

##### Materials

- **Type status:** Other material. **Occurrence:** catalogNumber: CIRR-305; recordedBy: Salvador Zarco Perello; individualCount: 10; **Location:** continent: America; country: Mexico; stateProvince: Yucatan; locality: Madagascar Reef; verbatimDepth: 16 m; verbatimLatitude: 781272.611854; verbatimLongitude: 2373523.69326; verbatimCoordinateSystem: UTM 15N; verbatimSRS: WGS84; decimalLatitude: 21.442191; decimalLongitude: -90.286277; **Event:** samplingProtocol: Photosampling; eventDate: 28/9/2007; **Record Level:** collectionID: YUC-PEC_239-01-64; institutionCode: UMDI-SISAL; collectionCode: CIRR

##### Distribution

Western Atlantic. North Carolina to south Florida and Gulf of Mexico.

#### Chaetodipterus faber

(Broussonet, 1782)

##### Materials

- **Type status:** Other material. **Occurrence:** recordedBy: Gabriela Martinez Portilla; individualCount: 1; **Location:** continent: America; country: Mexico; stateProvince: Yucatan; locality: Madagascar Reef; verbatimLatitude: 781272.611854; verbatimLongitude: 2373443.69326; verbatimCoordinateSystem: UTM 15N; verbatimSRS: WGS84; decimalLatitude: 21.441469; decimalLongitude: -90.286290; **Event:** samplingProtocol: Visual census; eventDate: 13/5/2005

##### Distribution

Western Atlantic. Massachusetts to South Brazil. Including Bermuda, Bahamas, and throughout the Caribbean Islands.

##### Notes

First record on a Campeche Bank reef. Occurrence reported by Martínez de la Portilla (2008).

#### Acanthurus tractus

Poey, 1860

##### Materials

- **Type status:** Other material. **Occurrence:** recordedBy: Gabriela Martinez Portilla; individualCount: 1; **Location:** continent: America; country: Mexico; stateProvince: Yucatan; locality: Madagascar Reef; verbatimLatitude: 781272.611854; verbatimLongitude: 2373443.69326; verbatimCoordinateSystem: UTM 15N; verbatimSRS: WGS84; decimalLatitude: 21.441469; decimalLongitude: -90.286290; **Event:** samplingProtocol: Visual census; eventDate: 13/5/2005

##### Distribution

Western Atlantic. Massachusetts to Brazil. Including Bermuda, Bahamas, and throughout the Caribbean Islands.

##### Notes

Occurrence reported by Martínez de la Portilla (2008).

#### Acanthurus coeruleus

Bloch & Schneider, 1801

##### Materials

- **Type status:** Other material. **Occurrence:** catalogNumber: CIRR-285; recordedBy: Salvador Zarco Perello; individualCount: 4; **Location:** continent: America; country: Mexico; stateProvince: Yucatan; locality: Madagascar Reef; verbatimDepth: 5 m; verbatimLatitude: 780535.103072; verbatimLongitude: 2373608.16789; verbatimCoordinateSystem: UTM 15N; verbatimSRS: WGS84; decimalLatitude: 21.443068; decimalLongitude: -90.293373; **Event:** samplingProtocol: Photosampling; eventDate: 20/9/2007; **Record Level:** collectionID: YUC-PEC_239-01-64; institutionCode: UMDI-SISAL; collectionCode: CIRR

##### Distribution

Western Atlantic. New York to Brazil. Including Bermuda, Bahamas, and throughout the Caribbean Islands.

#### Sphyraena barracuda

(Edwards, 1771)

##### Materials

- **Type status:** Other material. **Occurrence:** catalogNumber: CIRR-301; recordedBy: Salvador Zarco Perello; individualCount: 3; **Location:** continent: America; country: Mexico; stateProvince: Yucatan; locality: Madagascar Reef; verbatimDepth: 4 m; verbatimLatitude: 781272.611854; verbatimLongitude: 2373443.69326; verbatimCoordinateSystem: UTM 15N; verbatimSRS: WGS84; decimalLatitude: 21.441469; decimalLongitude: -90.286290; **Event:** samplingProtocol: Photosampling; eventDate: 28/9/2007; **Record Level:** collectionID: YUC-PEC_239-01-64; institutionCode: UMDI-SISAL; collectionCode: CIRR

##### Distribution

Worldwide.

#### Scomberomorus maculatus

(Mitchill, 1815)

##### Materials

- **Type status:** Other material. **Occurrence:** catalogNumber: CIRR-307; recordedBy: Salvador Zarco Perello; individualCount: 2; **Location:** continent: America; country: Mexico; stateProvince: Yucatan; locality: Madagascar Reef; verbatimDepth: 5 m; verbatimLatitude: 780535.103072; verbatimLongitude: 2373608.16789; verbatimCoordinateSystem: UTM 15N; verbatimSRS: WGS84; decimalLatitude: 21.443068; decimalLongitude: -90.293373; **Event:** samplingProtocol: Photosampling; eventDate: 20/9/2007; **Record Level:** collectionID: YUC-PEC_239-01-64; institutionCode: UMDI-SISAL; collectionCode: CIRR

##### Distribution

Western Atlantic. Maine to Yucatan, Gulf of Mexico.

#### Aluterus scriptus

(Osbeck, 1765)

##### Materials

- **Type status:** Other material. **Occurrence:** catalogNumber: CIRR-286; recordedBy: Salvador Zarco Perello; individualCount: 1; **Location:** continent: America; country: Mexico; stateProvince: Yucatan; locality: Madagascar Reef; verbatimDepth: 7 m; verbatimLatitude: 780143.766831; verbatimLongitude: 2373680.0708; verbatimCoordinateSystem: UTM 15N; verbatimSRS: WGS84; decimalLatitude: 21.443778; decimalLongitude: -90.297133; **Event:** samplingProtocol: Photosampling; eventDate: 24/9/2007; **Record Level:** collectionID: YUC-PEC_239-01-64; institutionCode: UMDI-SISAL; collectionCode: CIRR

##### Distribution

Worldwide.

## Discussion

Our study found that the fish fauna of Madagascar Reef consists of 8 orders, 30 families, 43 genera and 54 species. The families with the highest representation were: Serranidae (7), Gobiidae (5), Scaridae (4), Lutjanidae (3), Haemulidae (3) and Pomacanthidae (3) (Table 1). Almost all the species found at Madagascar Reef are distributed generally within the Gulf of Mexico (Felder and Camp 2009). However, *Hypoplectrus ecosur* was recorded here for the first time in the Gulf of Mexico, although it is possible that previous studies in the region have callected this species and identified as other species within the same genus (Victor 2012). *Elacatinus xanthiprora* was recorded here for the second time in Mexico, expanding its distribution westwards from Alacaranes Reef (González-Gandara and Arias-González 2001). *Sanopus reticulatus*, which is endemic to the Yucatan state, was recorded here for the first time at a reef, expanding its distribution westwards from Puerto Progreso (Collette 1983). Additionally, *Mycteroperca microlepis*, *Equetus lanceolatus* and *Chaetodipterus faber* are new species records for the CB reefs.

**Figure 6.**
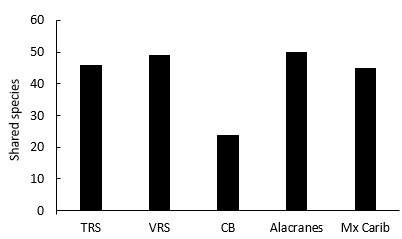
Number of species shared between Madagascar Reef and the reef systems of the Mexican Atlantic. TRS: Tuxpan Reef System; VRS: Veracruz Reef System; CB: Campeche Bank; Mx Carib: Mexican Caribbean. (Suppl. material 1).

The species composition found at Madagascar Reef was a mixture of species from other reef systems within the Mexican Atlantic (Figs 3, 4, 5). Among the species found at Madagascar Reef, 40 (74%) are registered for all of the reef systems of the Mexican Atlantic. The remaining 14 species (26%) are absent in at least one reef system. Madagascar Reef shared 45 (83%) species with TRS (González-Gándara et al. 2013), 48 (89%) with VRS (Del Moral Flores et al. 2013), 50 (91%) with Alacranes Reef (González-Gandara and Arias-González 2001), 24 (46%) with Arcas, Arenas and Triangulo Oeste reefs in the CB (Tunnell Jr et al. 2007) and 45 (85%) species with the Mexican Caribbean reefs (Schmitter-Soto et al. 2000) (Table 1, Fig. 6). *Mycteroperca microlepis* is shared only with the TRS and VRS and *Astrapogon stellatus* and *Hypoplectrus ecosur* are only shared with the Caribbean. Finally, *Elacatinus xanthiprora* is registered only in the CB and *Sanopus reticulatus* only in Madagascar reef (Table 1).

**Figure 7.**
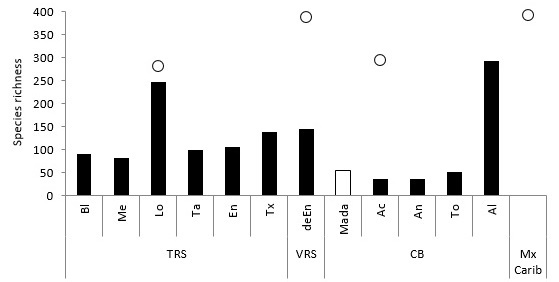
Species richness of coral reefs of the Gulf of Mexico and Mexican Caribbean (Mx Carib). Species number by individual reefs (bars) and reef systems (circles). Reef abbreviations: **TRS**: Tuxpan Reef System, Bl: Blanquilla, Me: Medio, Lo: Lobos, Ta: Tanhuijo, En: Enmedio, Tx: Tuxpan; **VRS**: Veracruz Reef System: deEn: De Enmedio; **CB**: Campeche Bank, Mada: Madagascar, Ac: Arcas, An: Arenas, To: Triangulo Oeste, Al: Alacranes. References: TRS: González-Gándara et al. 2013. VRS: Del Moral Flores et al. 2013; Tunnell Jr et al. 2007. CB: Tunnell Jr et al. 2007; Martínez de la Portilla 2008; and this work. Mexican Caribbean: Schmitter-Soto et al. 2000. (Suppl. material 2).

The species richness at Madagascar Reef is similar to other reefs in the CB, but lower than richness values reported for reefs in the VRS and TRS (Fig. 7). Of the four reefs in the CB with ichthyological information, three present lower species richness than Madagascar Reef. Cayos Arcas and Arenas have 37 species registered, whereas Triangulos Oeste has 52 (Tunnell Jr et al. 2007). Only Alacranes reef surpasses these numbers with 294 species (González-Gandara and Arias-González 2001). All the reefs in the TRS present higher richness. For example, Medio Reef has the lowest with 83 and Lobos Reef has the highest with 248 species, while each of the remaining reefs have approximately 100 species (González-Gándara et al. 2013). Similarly, Enmedio Reef in the SAV has 145 species (Tunnell Jr et al. 2007).

**Figure 8.**
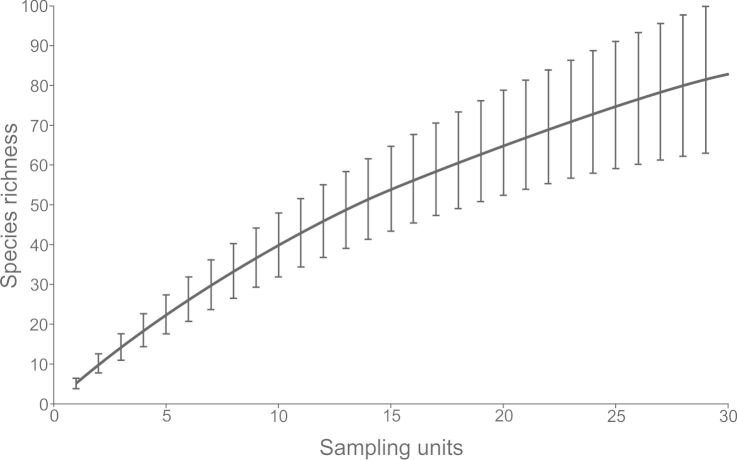
Fish species accumulation curve for Madagascar Reef. Calculated using sample-based incidence data with the software EstimateS v9 (http://purl.oclc.org/estimates) using Eq. 5 in Colwell et al. (2004) and Eq. 17 in Colwell et al. (2012) for rarefaction and Eq. 18 in [Bibr B668745][Bibr B668745] for extrapolation. Bars represent 95% confidence intervals calculated using Eq. 6 in Colwell et al. (2004) for rarefaction and Eq. 19 in Colwell et al. (2012) for extrapolation. For a full output of the analysis see Suppl. material 3.

The estimation of the species richness through the species accumulation curve clearly shows that the list of species presented here is not an exhaustive compendium of the species inhabiting this reef and that more species remain undiscovered (Fig. 8). With only three sampling campaigns comprising about 15 surveys (including Martínez de la Portilla (2008)) Madagascar Reef presented about 1/7 of the reef fish species in the Gulf of Mexico (376 species) (Withers and Tunnell Jr 2007). This numbers are lower than other better studied reefs but still quite significant given the low coral cover in the reef (Zarco-Perelló et al. 2013). The implementation of more studies at Madagascar Reef and other reefs in the CB not only would probably increase its species richness, reflecting the fish biodiversity of nearby hotspots such as Alacranes Reef (González-Gandara and Arias-González 2001) and the Caribbean (Schmitter-Soto et al. 2000) but also has the potential to extend distribution ranges of species from the Gulf of Mexico and Caribbean by finding new species records in this region, as was the case in the present study.

## Supplementary Material

Supplementary material 1 Fish species shared between Madagascar Reef and other Mexican Atlantic reefs and reef systems Data type: Spreadsheet
Brief description: Information of the fish species found in Madagascar Reef that are present/absent in other Mexican Atlantic reefs
File: oo_6998.csv Salvador Zarco-Perello

Supplementary material 2 Fish species richness of coral reefs of the Mexican Atlantic Data type: Spreadsheet
Brief description: Species richness of coral reefs of the Gulf of Mexico and Mexican Caribbean. Species number by individual reefs and reef systems.
File: oo_6999.csv Salvador Zarco-Perello

Supplementary material 3 Species richness estimation output Data type: text
File: oo_6959.txt Salvador Zarco-Perello

XML Treatment for Urobatis jamaicensis

XML Treatment for Gymnothorax funebris

XML Treatment for Synodus

XML Treatment for Sanopus reticulatus

XML Treatment for Holocentrus adscensionis

XML Treatment for Scorpaena plumieri

XML Treatment for Epinephelus adscensionis

XML Treatment for Epinephelus morio

XML Treatment for Hypoplectrus ecosur

XML Treatment for Mycteroperca bonaci

XML Treatment for Mycteroperca microlepis

XML Treatment for Mycteroperca venenosa

XML Treatment for Serranus subligarius

XML Treatment for Opistognathus aurifrons

XML Treatment for Astrapogon stellatus

XML Treatment for Echeneis

XML Treatment for Carangoides ruber

XML Treatment for Lutjanus apodus

XML Treatment for Lutjanus griseus

XML Treatment for Ocyurus chrysurus

XML Treatment for Anisotremus virginicus

XML Treatment for Haemulon aurolineatum

XML Treatment for Haemulon plumierii

XML Treatment for Calamus

XML Treatment for Equetus lanceolatus

XML Treatment for Pareques umbrosus

XML Treatment for Chaetodon ocellatus

XML Treatment for Holacanthus bermudensis

XML Treatment for Holacanthus ciliaris

XML Treatment for Pomacanthus arcuatus

XML Treatment for Abudefduf saxatilis

XML Treatment for Stegastes variabilis

XML Treatment for Kyphosus sectatrix

XML Treatment for Halichoeres

XML Treatment for Lachnolaimus maximus

XML Treatment for Thalassoma bifasciatum

XML Treatment for Scarus coeruleus

XML Treatment for Sparisoma aurofrenatum

XML Treatment for Sparisoma rubripinne

XML Treatment for Sparisoma viride

XML Treatment for Malacoctenus triangulatus

XML Treatment for Parablennius marmoreus

XML Treatment for Coryphopterus dicrus

XML Treatment for Coryphopterus glaucofraenum

XML Treatment for Elacatinus oceanops

XML Treatment for Elacatinus xanthiprora

XML Treatment for Tigrigobius macrodon

XML Treatment for Ptereleotris calliura

XML Treatment for Chaetodipterus faber

XML Treatment for Acanthurus tractus

XML Treatment for Acanthurus coeruleus

XML Treatment for Sphyraena barracuda

XML Treatment for Scomberomorus maculatus

XML Treatment for Aluterus scriptus

## Figures and Tables

**Figure 3a.**
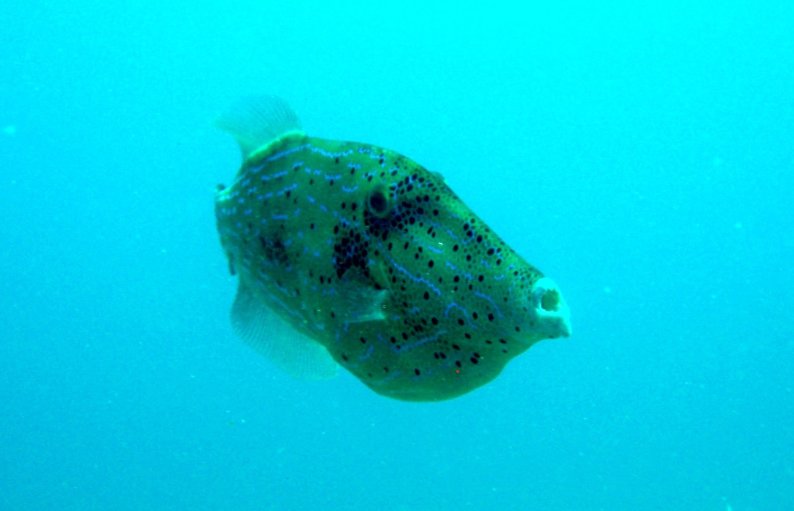
Aluterus scriptus

**Figure 3b.**
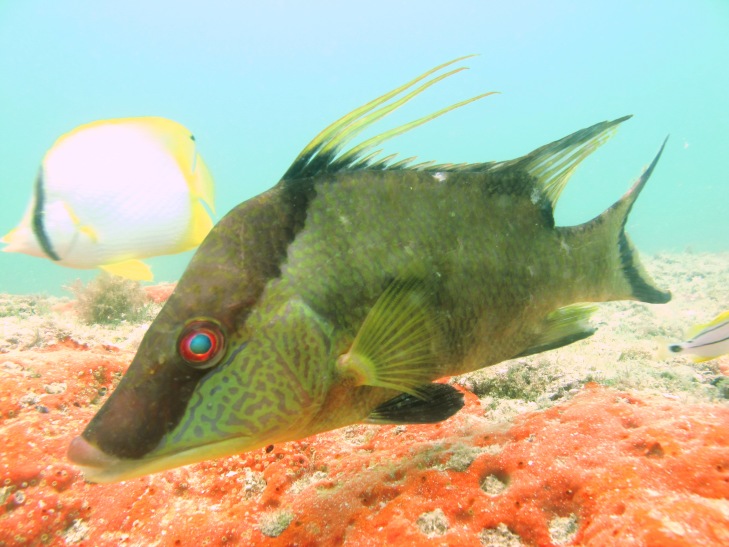
Lachnolaimus maximus

**Figure 3c.**
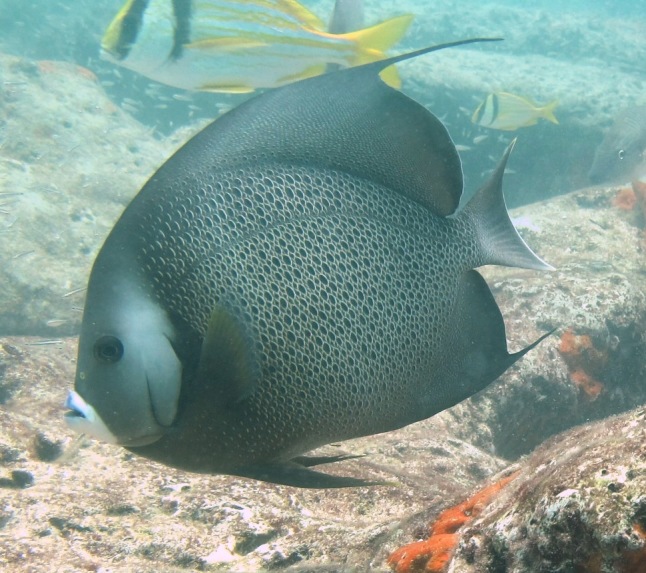
Pomacanthus arcuatus

**Figure 3d.**
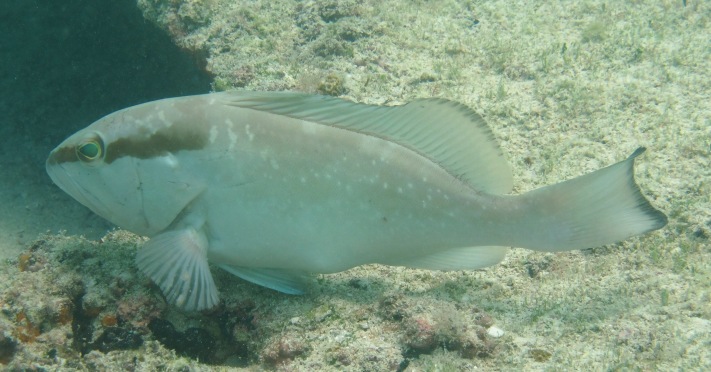
Epinephelus morio

**Figure 3e.**
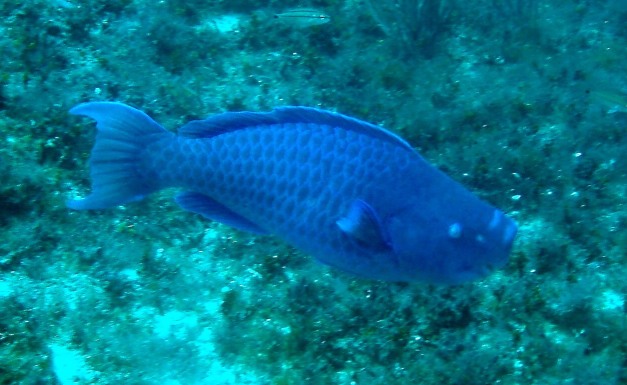
Scarus coeruleus

**Figure 3f.**
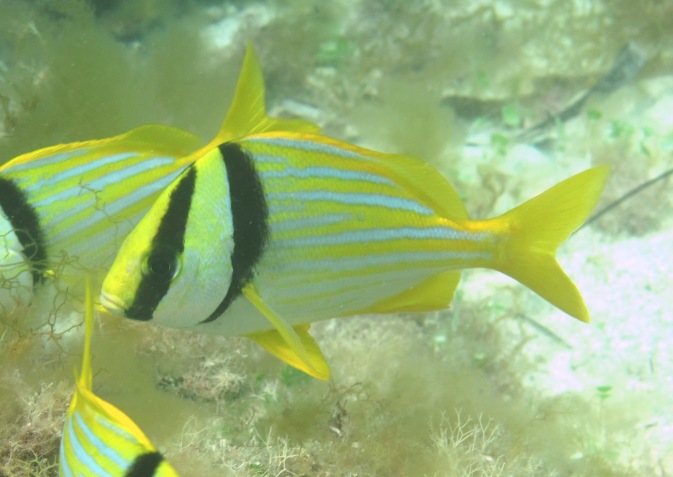
Anisotremus virginicus

**Figure 4a.**
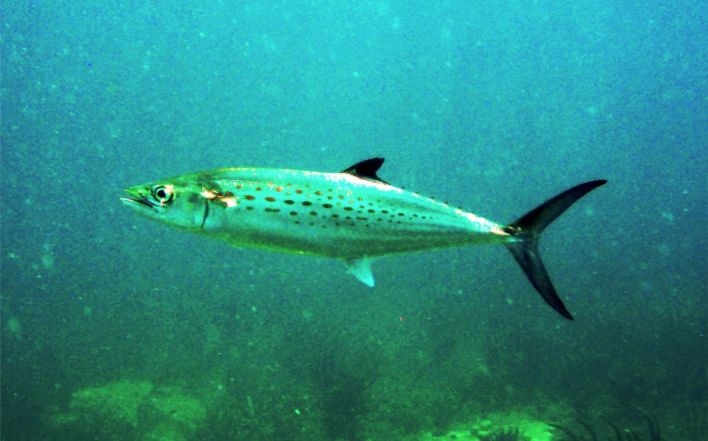
Scomberomorus maculatus

**Figure 4b.**
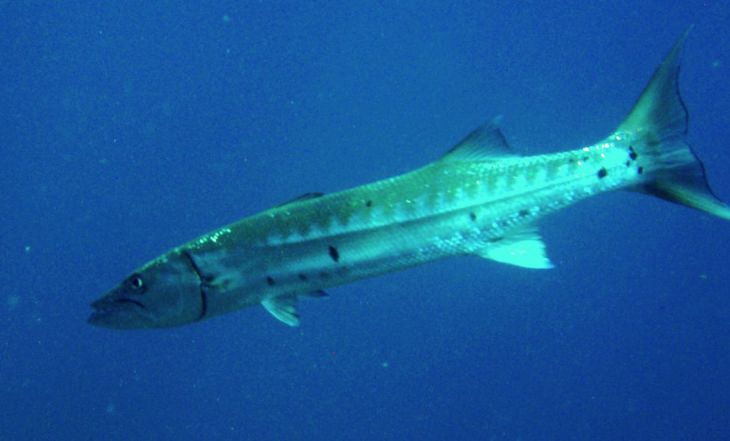
Sphyraena barracuda

**Figure 4c.**
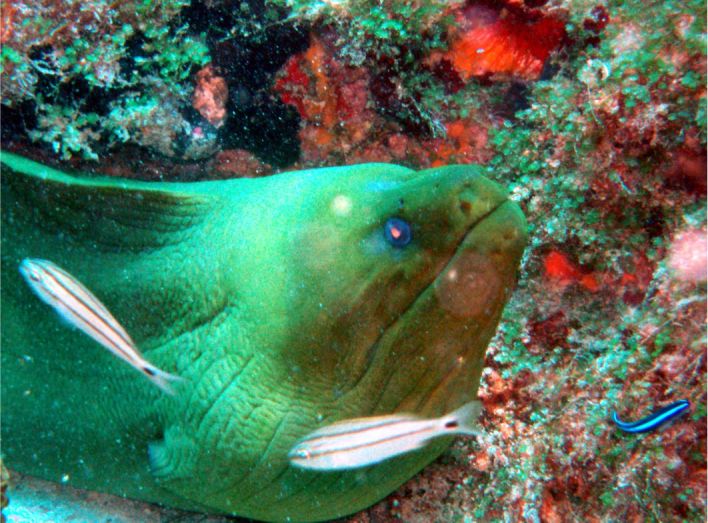
Gymnothorax funebris

**Figure 4d.**
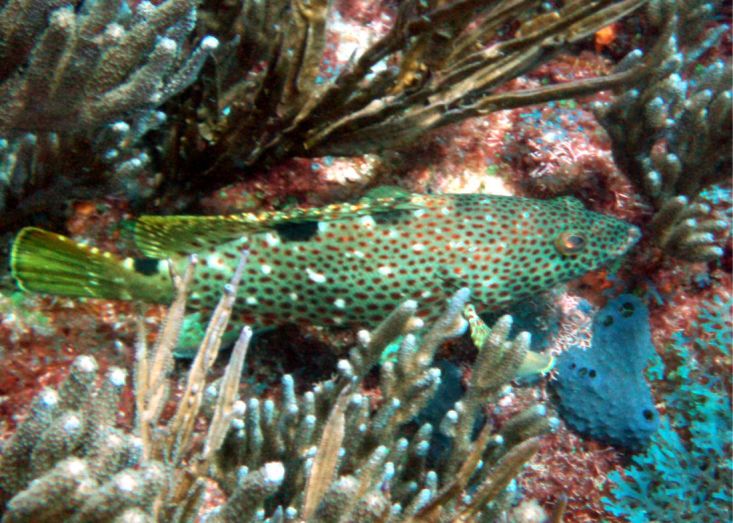
Epinephelus adscensionis

**Figure 4e.**
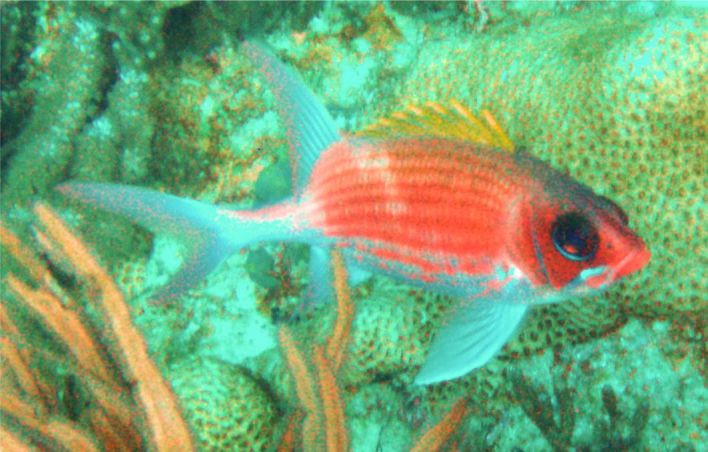
Holocentrus adscensionis

**Figure 4f.**
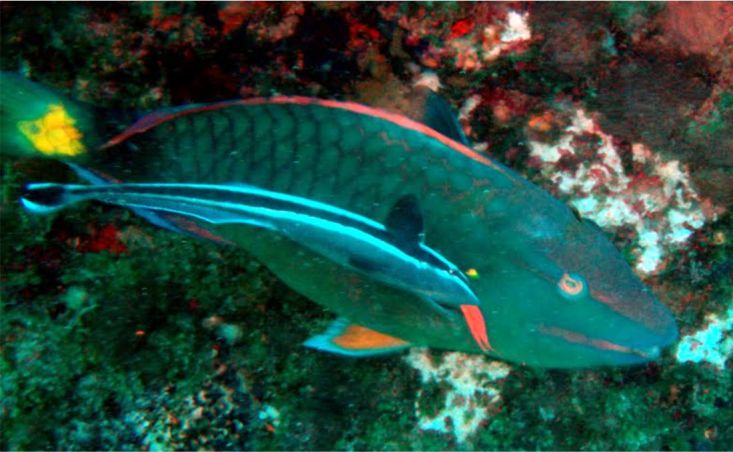
*Sparisoma viride* and *Echeneis* sp.

**Figure 5a.**
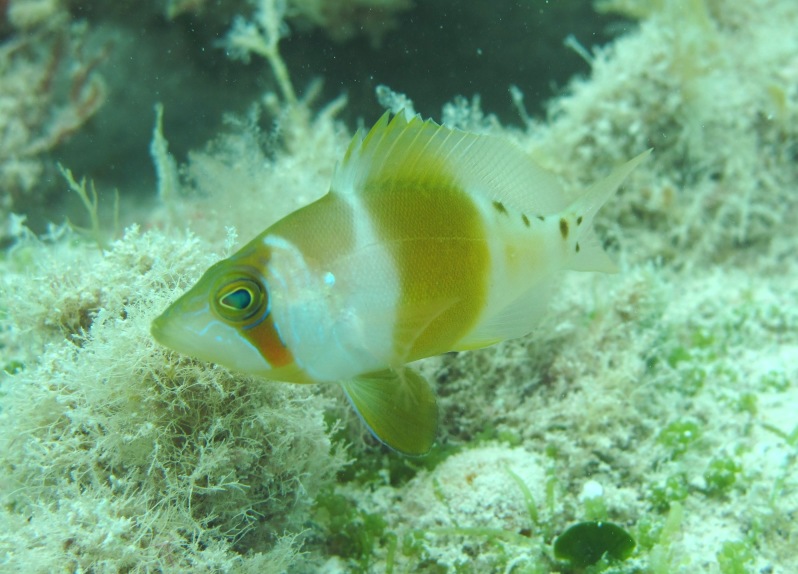
Hypoplectrus ecosur

**Figure 5b.**
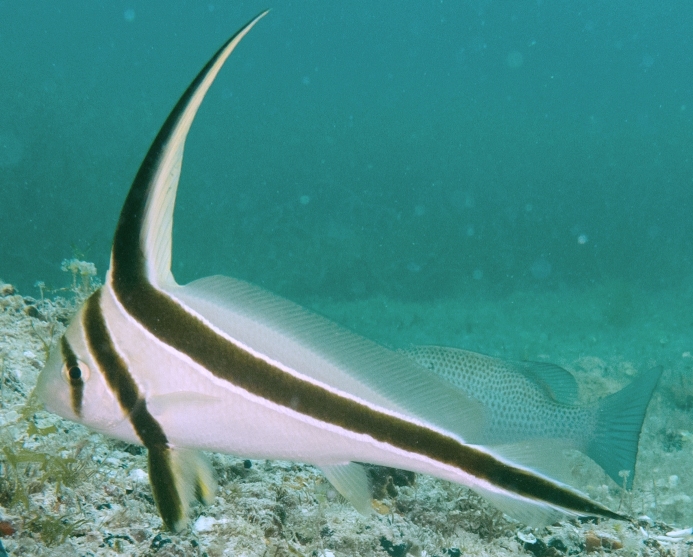
Equetus lanceolatus

**Figure 5c.**
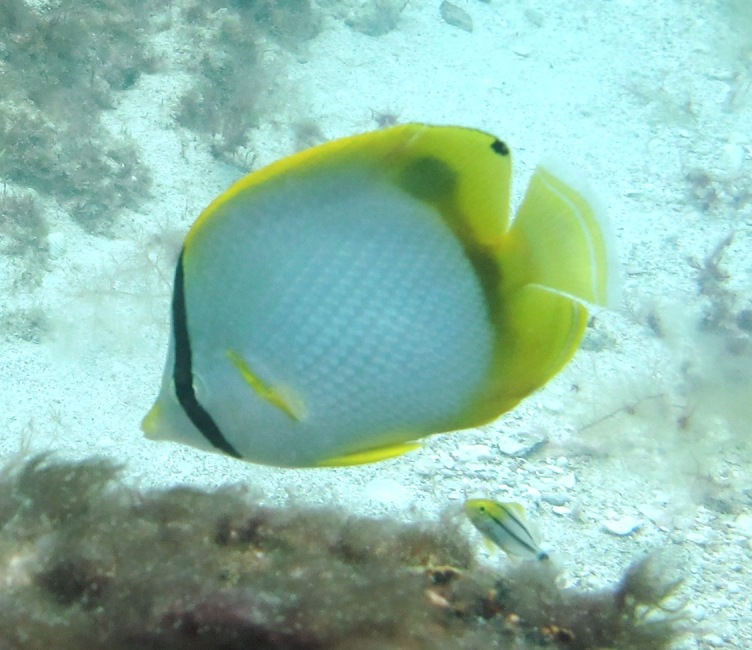
Chaetodon ocellatus

**Figure 5d.**
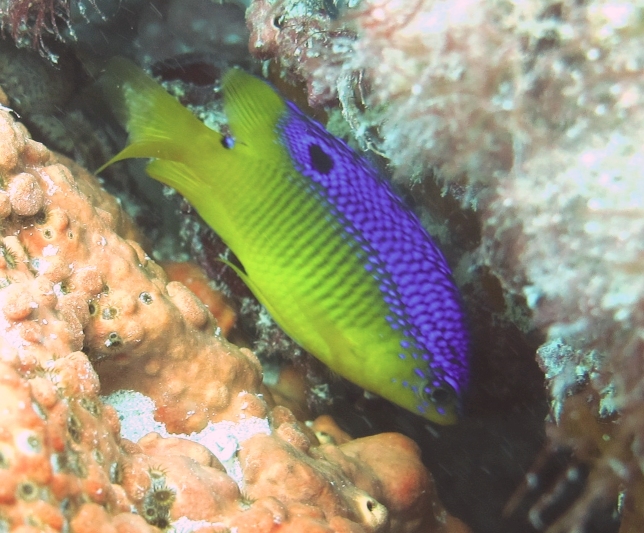
Stegastes variabilis

**Figure 5e.**
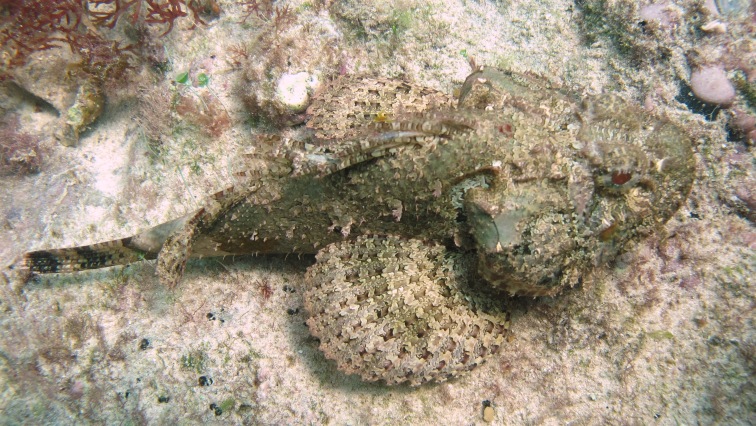
Scorpaena plumieri

**Figure 5f.**
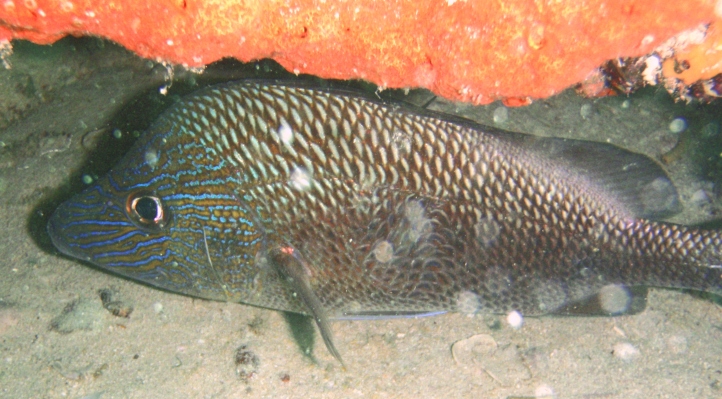
Haemulon plumierii

**Table 1.** Fish species of Madagascar Reef and its distribution on other reefs of the Gulf of Mexico. ID abbreviations: C: collected, Ph: Photograph, Vc: Visual census. Reef abbreviations: **TRS**: Tuxpan Reef System, Bl: Blanquilla, Me: Medio, Lo: Lobos, Ta: Tanhuijo, En: Enmedio, Tx: Tuxpan; **VRS**: Veracruz Reef System: dEn: De Enmedio; **CB**: Campeche Bank: Al: Alacranes, Ac: Arcas, Ar: Arenas, To: Triangulo Oeste, Ma: Madagascar; **Mx Carib**: Mexican Caribbean. The symbol • means present with no reef specified in the bibliography. References: (1) González-Gándara et al. 2013; (2) Del Moral Flores et al. 2013; (3) Tunnell Jr et al. 2007; (4) González-Gandara and Arias-González 2001; (5) Tuz-Sulub et al. 2006; (6) Taylor and Bright 1973; (7) Martínez de la Portilla 2008; (8) Schmitter-Soto et al. 2000; (9) This work.

Family	Genus and species	Authority	ID	TRS	VRS	CB	Mx Carib	References
Urotrygonidae	*Urobatis jamaicensis*	(Cuvier, 1816)	Ph	Tx	dEn	Al, Ma	•	1, 2, 4, 8, 9
Muraenidae	*Gymnothorax funebris*	Ranzani, 1839	Ph	Lo	dEn	Al, To, Ma	•	1, 2, 3, 4, 8, 9
Synodontidae	*Synodus* sp.		Vc	Lo, Ta, En, Tx	•	Al, Ma	•	1, 2, 4, 7, 8
Batrachoididae	*Sanopus reticulatus*	Collette, 1983	C			Ma		9
Holocentridae	*Holocentrus adscensionis*	(Osbeck, 1765)	Ph	Bl, Me, Lo, Ta, En, Tx	dEn	Al, To, Ma	•	1, 2, 3, 4, 8, 9
Scorpaenidae	*Scorpaena plumieri*	Bloch, 1789	C	Lo, Ta	dEn	Al, To, Ma	•	1, 2, 3, 4, 8, 9
Serranidae	*Epinephelus adscensionis*	(Osbeck, 1765)	Ph	Bl, Me, Lo, Ta, En, Tx	dEn	Al, Ac, Ar, To, Ma	•	1, 2, 3, 4, 6, 8, 9
	*Epinephelus morio*	(Valenciennes, 1828)	Ph		•	Al, Ma	•	2, 4, 8, 9
	*Hypoplectrus ecosur*	(Cuvier, 1828)	Ph			Ma		1, 2, 4, 8, 9
	*Mycteroperca bonaci*	(Poey, 1860)	Vc	En, Tx	dEn	Al, Ma	•	1, 2, 4, 7, 8
	*Mycteroperca microlepis*	(Goode & Bean, 1879)	Vc	Ta, En	•	Ma		1, 2, 9
	*Mycteroperca venenosa*	(Linnaeus, 1758)	Vc		dEn	Al, Ar, Ma	•	2, 3, 4, 5, 6, 7, 8
	*Serranus subligarius*	(Cope, 1870)	Vc	Ta, En, Tx	dEn	Al, Ma		1, 2, 4, 7
Opistognathidae	*Opistognathus aurifrons*	(Jordan & Thompson, 1905)	C		dEn	Al, Ma	•	2, 4, 8, 9
Apogonidae	*Astrapogon stellatus*	(Cope, 1867)	C			Al, Ma	•	4, 8, 9
Echeneidae	*Echeneis* sp.		Ph	Lo	dEn	Al, Ma	•	1, 2, 4, 8, 9
Carangidae	*Carangoides ruber* Syn. *Caranx ruber*	(Bloch, 1793) (Bloch, 1793)	Ph	Bl, Lo, Ta, En, Tx	dEn	Al, Ac, Ar, Ma	•	1, 2, 3, 4, 8, 9
Lutjanidae	*Lutjanus apodus*	(Walbaum, 1792)	Vc	Bl, Me, Lo, En, Tx	dEn	Al, Ac, Ar, Ma	•	1,2, 3, 4, 7, 8
Haemulidae	*Lutjanus griseus*	(Linnaeus, 1758)	Ph	Bl, Me, Lo, Ta, En, Tx	dEn	Al, To, Ma	•	1, 2, 3, 4. 8, 9
	*Ocyurus chrysurus*	(Bloch, 1791)	Ph	Bl, Me, Lo, Ta, En, Tx	dEn	Al, Ac, Ar, Ma	•	1, 2, 3, 4, 8, 9
	*Anisotremus virginicus*	(Linnaeus, 1758)	Ph	Bl, Me, Lo, Ta, En, Tx	dEn	Al, Ac, Ar, Ma	•	1, 2, 3, 4, 8, 9
	*Haemulon aurolineatum*	Cuvier, 1830	Ph	Bl, Me, Lo, Ta, En, Tx	dEn	Al, Ac, Ar, Ma	•	1, 2, 3, 4, 8, 9
	*Haemulon plumierii*	(Lacepède, 1801)	Ph	Bl, Me, Lo, Ta, En, Tx	•	Al, Ac, Ar, Ma	•	1, 2, 3, 4, 7, 8, 9
Sparidae	*Calamus* sp.		Ph	Bl, Me, Lo, Ta, En, Tx	dEn	Al, Ma	•	1, 2, 4, 8, 9
Scianidae	*Equetus lanceolatus*	(Linnaeus, 1758)	Ph	Lo, Tx	dEn	Ma	•	1, 2, 4, 8, 9
	*Pareques umbrosus*	(Jordan & Eigenmann, 1889)	Ph	En	•	Al, Ma	•	1, 2, 4, 8, 9
Chaetodontidae	*Chaetodon ocellatus*	Bloch, 1787	Ph	Bl, Me, Lo, Ta, En, Tx	dEn	Al, To, Ma	•	1, 2, 3, 4, 7, 8, 9
Pomacanthidae	*Holacanthus bermudensis*	Goode, 1876	Ph	Bl, Me, Lo, En, Tx	•	Al, Ma	•	1, 2, 4, 8, 9
	*Holacanthus ciliaris*	(Linnaeus, 1758)	Vc	Bl, Me, Lo, Tx	dEn	Al, Ma	•	1, 2, 4, 7, 8
	*Pomacanthus arcuatus*	(Linnaeus, 1758)	Ph		dEn	Al, Ma	•	2, 4, 8, 9
Pomacentridae	*Abudefduf saxatilis*	(Linnaeus 1758)	Ph	Bl, Me, Lo, Ta, En, Tx	dEn	Al, Ac, Ar, To, Ma	•	1, 2, 3, 4, 8, 9
	*Stegastes variabilis*	(Castelnau, 1855)	Ph	Bl, Me, Lo, Ta, En, Tx	dEn	Al, Ac, Ar, To, Ma	•	1, 2, 3, 4, 8, 9
Kyphosidae	*Kyphosus sectatrix*	(Linnaeus 1758)	Vc	Bl, Lo, Ta, Tx	dEn	Al, To, Ma	•	1, 2, 3, 4, 7, 8
Labridae	*Halichoeres* sp.		Vc	Bl, Me, Lo, Ta, En, Tx	dEn	Al, Ac, Ar, To, Ma	•	1, 2, 3, 4, 7, 8
	*Lachnolaimus maximus*	(Walbaum, 1792)	Ph	Bl, Lo, Tx	dEn	Al, Ma	•	1, 2, 4, 8, 9
	*Thalassoma bifasciatum*	(Bloch, 1791)	Vc	Bl, Me, Lo, Ta, En, Tx	dEn	Al, Ac, Ar, To, Ma	•	1, 2, 3, 4, 7, 8
Scaridae	*Scarus coeruleus*	(Edwards, 1771)	Ph	Me, Lo, Ta	dEn	Al, Ac, Ar, Ma	•	1, 2, 3, 4, 8, 9
	*Sparisoma aurofrenatum*	(Valenciennes, 1840)	Ph	Bl, Me, Lo, Ta, En, Tx	•	Al, Ma	•	1, 2, 4, 8, 9
	*Sparisoma rubripinne*	(Valenciennes, 1840)	Ph	Bl, Me, Lo, Ta, En, Tx	dEn	Al, To, Ma	•	1, 2, 3, 4, 8, 9
	*Sparisoma viride*	(Bonnaterre, 1788)	Ph	Bl, Me, Lo, Ta, En, Tx	dEn	Al, Ac, Ar, To, Ma	•	1, 2, 3, 4, 8, 9
Labrisomidae	*Malacoctenus triangulatus*	Springer, 1959	C	Bl, Me, Lo, Ta, En, Tx	•	Al, Ma	•	1, 2, 4, 8, 9
Blennidae	*Parablennius marmoreus*	(Poey, 1876)	C	Bl, Me, Lo, En, Tx	dEn	Al, Ma		1, 2, 4, 9
Gobiidae	*Coryphopterus dicrus*	Böhlke & Robins, 1960	C	Lo	•	Al, Ma	•	1, 2, 4, 8, 9
	*Coryphopterus glaucofraenum*	Gill, 1863	C	Bl, Me, Lo, Ta, En, Tx	•	Al, Ma	•	1, 2, 4, 8, 9
	*Elacatinus oceanops*	Jordan, 1904	C, Ph	Me, Lo, Ta, En, Tx	•	Al, Ma	•	1, 2, 4, 8, 9
	*Elacatinus xanthiprora*	(Böhlke & Robins, 1968)	C			Al, Ma		4, 9
	*Tigrigobius macrodon* Syn. *Elacatinus macrodon* *Gobiosoma macrodon*	(Beebe & Tee-Van, 1928) (Beebe & Tee-Van, 1928) Beebe & Tee-Van, 1928	Vc	Lo		Ma		1, 9
Microdesmidae	*Ptereleotris calliura*	(Jordan & Gilbert, 1882)	Ph	Tx		Al, Ma		1, 4, 9
Ephippidae	*Chaetodipterus faber*	(Broussonet, 1782)	Vc	Lo, Ta, Tx	•	Ma	•	1, 2, 7, 8
Acanthuridae	*Acanthurus tractus* Syn. *Acanthurus bahianus*	Poey, 1860 Castelnau, 1855	Vc	Bl, Me, Lo, Ta, En, Tx	dEn	Al, Ac, Ar, To, Ma	•	1,2, 3, 4, 7, 8
	*Acanthurus coeruleus*	Bloch & Schneider, 1801	Ph	Bl, Me, Lo, Ta, En, Tx	dEn	Al, Ac, Ar, To, Ma	•	1, 2, 3, 4, 8, 9
Sphyraenidae	*Sphyraena barracuda*	(Edwards, 1771)	Ph	Bl, Lo, Ta, En, Tx	dEn	Al, Ma	•	1, 2, 4, 8, 9
Scombridae	*Scomberomorus maculatus*	(Mitchill, 1815)	Ph		dEn	Al, Ma		2, 4, 9
Monacanthidae	*Aluterus scriptus*	(Osbeck, 1765)	Ph	Lo, Tx	dEn	Al, Ma	•	1, 2, 4, 8, 9
